# Determinants of tuberculosis treatment support costs to the treatment supporters in rural Ghana

**DOI:** 10.3934/publichealth.2023007

**Published:** 2023-02-21

**Authors:** Robert Bagngmen Bio, Patricia Akweongo, Augustine Adomah-Afari, Augustina Koduah

**Affiliations:** 1 School of Public Health, University of Ghana, Legon, Accra, Ghana; 2 School of Pharmacy, University of Ghana, Legon, Accra, Ghana

**Keywords:** Bono region, direct cost, indirect cost, Ghana, tuberculosis, treatment supporter

## Abstract

**Background:**

The Ghana Health Service has been implementing the Directly Observed Therapy Short Course (DOTS) strategy for decades now, to cure and reduce the transmission of tuberculosis. DOTS strategy requires TB patients and their treatment supporters to make multiple clinic visits in the course of treatment, and this may place financial burden on treatment supporters with low socio-economic status. However, the determinants of tuberculosis treatment support costs to treatment supporters are unknown in Ghana.

**Objectives:**

This study determined the costs associated with treatment support to the treatment supporters in Bono Region, Ghana.

**Methods:**

In a cross-sectional study using cost-of-illness approach, 385 treatment supporters were selected and interviewed. A validated questionnaire for the direct and indirect costs incurred was used. Descriptive statistics and bivariate techniques were used for data analysis.

**Results:**

Averagely, each treatment supporter spent GHS 122.4 (US$ 21.1) on treatment support activities per month, which is about 19% of their monthly income. The findings also revealed that highest level of education, household size, monthly income and district of residence were significant predictors of the direct costs. On the other hand, gender of the respondents, highest level of education, ethnicity, household size, income level and relationship with patient were some of the factors that significantly influenced the indirect costs. The significance levels were set at a 95% confidence interval and p < 0.05.

**Conclusion:**

The study concludes that the estimated cost associated with assisting tuberculosis patients with treatment is significant to treatment supporters. If these costs are not mitigated, they have the tendency of affecting the socio-economic status and welfare of individuals assisting tuberculosis patients with treatment.

## 1. Introduction

Tuberculosis (TB) is a common cause for mortality and is highly prevalent especially in most developing countries [1]. In Ghana, the incidence of tuberculosis is generalized, affecting every region, district and community. It is estimated that 46,000 new cases of tuberculosis occur each year in Ghana [2]. WHO estimates that Ghana is detecting only 26% of all TB cases, which is well below the African regional average of 47% and WHO's target of 85% [3]. This is against the background that drugs that can treat TB have been discovered over 50 years ago [4]. Generally, involving treatment supporters as part of DOTS strategy has been widely found to be very essential to TB control [1],[5].

However, elimination of tuberculosis treatment associated costs to all parties involved has been very challenging [6]. This has necessitated the post-2015 global strategy to control tuberculosis aiming at identifying and eliminating all tuberculosis treatment associated costs to all parties involved by 2025 [7]. The financial impact of assisting tuberculosis patients with treatment has received little or no attention in policies for tuberculosis control.

Despite the financial implications of providing treatment support, there are limited published empirical studies quantifying the economic burden of tuberculosis to the treatment supporters globally. While there are published economic burden studies that have estimated the costs of tuberculosis in both developed and developing countries, the emphasis has generally been on the health system burden, the tuberculosis patient and their households and not the cost incurred by individuals who are providing support to the tuberculosis patient [8]–[10].

As far as we are aware, we are the first to estimate the direct and indirect costs of TB treatment support in a comprehensive way.

## 2. Materials and methods

This study was a cross-sectional analytical study using the quantitative approach of data collection.

### 2.1. Study setting

This study was conducted in six sites in the Bono Region, including three districts and three municipalities in the region. These included Berekum Municipality, Dormaa Municipality, Sunyani Municipality, Jaman North District, Jaman South District and Tain District. The Jaman North District, Jaman South District and Tain District are rural, as compared to the Berekum Municipality, Dormaa Municipality and Sunyani Municipality, which are urbanized.

The selection of these districts/municipalities was based on prevalence of tuberculosis. In addition, the background analysis of the study districts showed similar economic and demographic equivalence that allowed inferences to be made to cover the entire region. Farming is the major economic driving force of the region.

### 2.2. Healthcare delivery

Healthcare delivery in the region also follows the decentralized structure: regional, district, sub-district and community level [11]. The Bono region is made up of 11 administrative districts. The region has a total of 212 health facilities, comprising 1 regional hospital (located at the regional capital-Sunyani), 9 district hospitals, 82 health centers, 56 clinics, 35 private maternity homes and 112 functional Community-based Health Planning and Services (CHPS) and several other demarcated CHPS dotted across the region. Two out of the 11 districts do not have district hospitals-Banda and Sunyani West districts [11]. These health facilities render both preventive and curative healthcare services to the population within their catchment areas [11].

Diagnosis and treatment of TB across the country is provided based on an adopted national guideline that describes diagnostic and treatment standards [12]. Thus, diagnosis of TB is made using sputum smear microscopy and clinical signs. Treatment regimens, on the other hand, are based on the adopted Directly Observed Treatment Strategy (DOTS) [12]. The national tuberculosis program coordinates all tuberculosis control-related activities in Ghana, including all tuberculosis control activities in the Bono Region.

### 2.3. Study population

The study population comprised adult TB treatment supporters who are 18 years and above residing in the Bono Region of Ghana.

#### 2.3.1. Inclusion and exclusion criteria

All TB treatment supporters who were 18 years and had observed TB patient treatment for at least two months were included in the study. All treatment supporters who have not provided treatment support for up to two months were excluded from the study.

#### 2.3.2. Sampling

Assuming that each tuberculosis patient had one treatment supporter, 385 of them were used for this study, assigned in proportion to the number of tuberculosis patients in each district using the formula $\frac{\text{number of treatment supporters in each district}}{\text{total number of treatment supporters in the six districts}}\times 385$.

**Table 1.** Number of tuberculosis patients in treatment as of 2018 and sample size allocated.

No	District	No. of TB Cases	Sample Size	%
1	Berekum Municipality	112	68	18
2	Dormaa Municipality	89	54	14
3	Jaman North	78	48	12
4	Jaman South	62	38	10
5	Sunyani Municipality	170	104	27
6	Tain	120	73	19
	TOTAL	631	385	100

#### 2.3.3. Selection of participants

Six district/municipal hospitals providing DOTS services were purposively selected for the study based on the top 6 districts with the highest number of TB cases. At each study site, treatment supporters who attended the hospital with TB patients for drug rations were identified. Those treatment supporters who agreed to participate in the study were interviewed until 385 respondents were obtained.

### 2.4. Description of costs variables

Detailed descriptions of the costs variables are given in Table 2.

**Table 2.** Descriptions of costs.

Cost Type	Cost Category	Description
Direct Cost	Feeding	Direct out-of-pocket payments for food and water.
	Travel/transportation	Cost associated with travel to and from the health facility for TB medications.
	Accommodation	Direct out-of-pocket payments for lodging.
	Other: communication	Direct out-of-pocket payments on mobile credit/phone calls.
Indirect Cost (productivity losses associated with providing TB treatment support)	Time spent on travel/transportation	Productive time spent on travel to and from health facility for TB medications.
	Time spent with TB patient	Productive time spent with TB patient supervising/serving TB medications.
	Waiting Time	Productive time spent at health facility waiting for TB medications.

### 2.5. Data collection

Instruments for data collection were developed in line with the objectives of the study. A standardized and structured questionnaire was used to solicit information about the socio-demographic characteristics of the respondents and the direct and indirect costs incurred by treatment supporters. Some of the key demographic characteristics captured included age of respondent in completed years, gender of respondent, level of educational attainment, ethnicity, religious affiliation, occupation of respondent, monthly income and household size, among others. The questionnaire also covered treatment support expenditures, which included transportation, food, lodging and communication under the direct costs and travel time to and from the health facility, time spent with TB patient and waiting time at the health facility under the indirect cost components.

### 2.6. Data analysis

Quantitative data analysis techniques were employed to make meaning of the data collected. Microsoft Excel 2013 and STATA 14 were the computer software tools used for the data management. Descriptive statistics and cost estimations techniques were employed. Also, bivariate analysis to establish socio-demographic factors influencing the costs of TB treatment support was performed using the one-way ANOVA test of equality of the mean difference. The significance level was set at a 95% confidence interval and a P-value < 0.05.

### 2.7. Costs estimation

The costs associated with TB treatment support constitute the resources that are spent on providing support to a TB patient in treatment. They also include the monetary value of productive time lost to perform TB treatment support-related activities. These costs were categorized into direct and indirect costs.

#### 2.7.1. Direct costs

The direct cost was the sum of all the out-of-pocket payments made for transportation, feeding, accommodation and communication.

#### 2.7.2. Indirect costs

The indirect cost associated with treatment support was the value of productive time lost to carry out TB treatment support activities. We estimated the indirect costs using the human capital approach. The human capital approach takes the individual's perspective, and it is based on the assumption that all productive time lost should be valued in monetary equivalence [13]. This assumption serves as the basis for the indirect cost estimation for this study.

Treatment supporters were requested to estimate time lost due to Directly Observed Therapy Short Course (DOTS) center visits for drug rations, waiting at the DOTS center and time spent with the TB patient observing treatment. The times in any units (seconds, minutes or hours) were converted to hours and then to days at an average of 8 working hours in a day (8 hours = 1 day), recommended by the Ghana Labor Commission [14].

The number of days was then multiplied by the 2019 daily minimum wage rate (GHS 10.65 = US$ 1.99) for treatment supporters who were employed in the formal sector. Due to the complexity of the informal market arrangement and data constraints, the average daily agricultural labor (“by-day”) wage rate (GHS 37.5 = US$ 7.03) in the Bono Region in November 2019 was used for the valuation of the indirect costs of treatment supporters in the informal sector. All the costs were inquired in local currency (Ghana Cedis) and then converted into US dollars (US$) using an average exchange rate of GHS 5.33 for US$ 1 (Available from: https://www.bog.gov.gh/economic-data/exchange-rate/), the interbank exchange rate in November 2019 for international comparison with other cost-of-illness studies.

### 2.8. Ethical considerations

The protocol for this study was reviewed and approved by the Ghana Health Service Ethics Review Committee with reference number GHS-ERC 003/03/2019.

## 3. Results

### 3.1. Socio-demographic characteristics of respondents

Results of the treatment supporters' socio-demographic characteristics are summarized in Table 3. The total number of respondents in this study was 385. The mean age of the treatment supporters was 39 years. The results showed that 90 (23%) of them were aged 25–34 years, 174 (45%) were aged 35–44 years, and 95 (25%) were aged 45–54 years. A total of 205 (53%) of the treatment supporters were male, and 180 (47%) of them were females. Also, 284 (74%) of the treatment supporters were married, 66 (17%) of them were not married, and 23 (6%) of the treatment supporters were widowed.

A total of 303 (79%) of the treatment supporters were Christians, and 82 (21%) of them were non-Christians. The results revealed that 146 (38%) of them completed junior high school, and 137 (36%) completed senior high school. In terms of ethnicity, 233 (61%) of the treatment supporters were Bonos, 77 (20%) were Akans, and 75 (19%) of them were from other ethnic groups of northern extraction. About 281 (77.4%) of the treatment supporters were employed in the informal sector, and 82 (22.6%) were employed in the formal sector.

About 148 (38%) of the treatment supporters earned GHS 500 – GHS 750, and 134 (35%) of them were earning < GHS 500. The average monthly income was GHS 520. In terms of relationships with the patients, about 276 (72%) of them were family members, and 75 (20%) were friends supporting the TB patients. The results revealed that 214 (56%) of the treatment supporters engaged in faming as their occupation, 67 (17%) of them engaged in trading, and 24 (6%) of them were engaged as government employees.

Regarding household size, less than half of the treatment supporters [166 (43.1%)] had a household size of <4 members, and nearly the same proportion [154 (40%)] of them were living in households with a size of 4–7 persons. A total of 104 (27%) of the respondents lived in Sunyani, the regional capital, 73 (19%) of them lived in Tain, and a minority [38 (9.9%)] lived in the Jaman South District.

**Table 3.** Socio-demographic characteristics of respondents (n = 385).

Characteristics	N	(%)
Age group		
18–24	13	3.4
25–34	90	23.4
35–44	174	45.2
45–54	95	24.7
>54	13	3.4
Gender		
Male	205	53.3
Female	180	46.8
Marital status		
Never Married	66	17.1
Married	284	73.8
Widowed	23	6.0
Divorced/Separated	12	3.1
Religion		
Christian	303	78.7
Non-Christian	82	21.3
Highest level of education		
Primary	69	17.9
Junior High School	146	37.9
Senior High School	137	35.6
Tertiary (University)	33	8.6
Ethnicity		
Akan	77	20.0
Bono	233	60.5
Dagaaba/ Frafra/ Mo	75	19.5
Household size		
Alone	22	5.7
<4	166	43.1
4–5	154	40.0
6–7	43	11.2
Type of occupation		
Farming	214	55.6
Government employee	24	6.2
Private employee	58	15.0
Trading	67	17.4
Students	6	1.6
Unemployed	16	4.2
Sector of employment (363)		
Formal sector	82	22.6
Informal sector	281	77.4
Monthly income		
No income	22	5.7
<500	134	34.8
500–750	148	38.4
750–999	34	8.8
>999	47	12.2
Relationship with patient		
Family member	276	71.7
Friend	75	19.5
Health worker	22	5.7
Spouse	12	3.1
District of Residence		
Berekum Municipal	68	17.7
Dormaa Municipal	54	14.0
Jaman North	48	12.5
Jaman South	38	9.9
Sunyani Municipal	104	27.0
Tain	73	19.0

### 3.2. Distribution of cost elements and time lost to treatment support work

Table 4 gives the breakdown of the cost items and productive time lost to tuberculosis treatment support activities. A total of 298 (77.4%) treatment supporters incurred costs for feeding. Feeding costs included direct out-of-pocket payments for food and water when treatment supporters visited the health facility.

Regarding transportation, 237 (61.6%) treatment supporters incurred costs from traveling to and from the health facility. The results revealed that 155 (29.9%) of the treatment supporters used motorbikes as a means of transport to and from the health facility, while 122 (31.8%) of them used public transport (taxis, minibuses) as their means of transport, and 148 (38.4%) went to and from the health facility by walking. Concerning costs of communication, 197 (51.2%) incurred costs for phone calls.

The results showed that the majority, 281 (77.4%), were employed in the informal sector of the economy, while 82 (22.4%) of them were employed in the formal sector of the economy.

A total of 5920.8 hours were lost by the 363 treatment supporters who were actively employed in caring for the TB patients. Treatment supporters in the formal sector lost 1151.1 productive hours to tuberculosis treatment support, while treatment supporters in the informal sector lost 4769.7 hours. In all, 330 (90.9%) of the treatment supporters who were economically active reported lost earnings. Of the 330 treatment supporters who reported lost earnings, the majority, 278 (84.2%), were working in the informal sector, and 52 (15.9%) of them were working in the formal sector.

**Table 4.** Distribution of cost elements and time lost to treatment support work.

Characteristic	N (%)	Time (Hours)
Proportion of treatment supporters that incurred costs for food (n = 385)	298 (77.4)	-
Proportion of treatment supporters that incurred costs for transport (n = 385)	237 (61.6)	-
Proportion of treatment supporters that incurred costs for communication (n = 385)	197 (51.2)	-
Type of Transport (n = 385)
Motorbikes	115 (29.9)	-
Public transport (taxi, mini-buses)	122 (31.7)	-
Walking (by foot)	148 (38.4)	-
Average number of visits to the facility per month (n = 385)	(2.1)	-
Productivity losses: Time loss (n = 363)
Formal Sector	82 (22.6)	1151.1
Informal Sector	281 (77.4)	4769.7
Total	363 (100)	5920.8
Average productive time lost (n = 363)	-	(16.3)
Productivity losses: lost earnings (n = 330)
Formal sector	52 (15.8)	-
Informal sector	278 (84.2)	-

### 3.3. Costs of TB treatment support to the treatment supporters

The average total cost of providing treatment support to the treatment supporters per month was GHS 112.5 (US$ 21.1). The key components of direct cost were transportation (about 17%) and feeding (15%), among the total costs. The results are displayed in Table 5.

**Table 5.** Costs of TB treatment support to the treatment supporters.

Cost component	N	Average cost (GHS)	Average cost (US$)	Cost profile (%)
Direct Cost
Food	298	17.8	3.3	14.6
Transportation	237	25.4	4.8	16.8
Others	197	3.7	0.7	2.1
Total direct cost		46.8	8.7	33.4
Indirect Cost
Formal Sector	82	18.7	3.5	4.3
Informal Sector	281	79.4	14.9	62.3
Total Indirect Cost	363	65.7	12.3	66.6

### 3.4. Bivariate analysis: Mean difference of TB treatment support costs by socio-demographic characteristics of respondents

Table 6 shows the one-way ANOVA test of equality of the mean direct, indirect and total costs of TB treatment support by socio-demographic characteristics of the study respondents. In terms of total direct cost of TB treatment support, the one-way ANOVA test showed that there were significant differences in the mean TB treatment support cost by religion (T = −2.2, p = 0.03), highest level of education (F = 9.0, p < 0.001), household size (F = 10.2, p < 0.001), monthly income (F = 5.7, p < 0.001) and district of residence (F = 6.7, p < 0.001).

Similarly, the mean total indirect cost of TB treatment support varied significantly by sex of respondents (F = 47.4, p < 0.001), marital status (F = 3.8, p = 0.01), religion (T = −8.4, p < 0.001), highest level of education (F = 37.5, p < 0.001), ethnicity (F = 7.9, p < 0.001), household size (F = 40.0, p < 0.001), employment sector (F = 404, p < 0.001), monthly income (F = 53.4, p < 0.001) and relationship with patient (F = 29.1, p < 0.001).

**Table 6.** Bivariate analysis: mean differences of TB treatment support costs by socio-demographic characteristics of respondents.

Characteristics	Total direct cost (GHS)		Total indirect cost (GHS)	
	Mean ± SD	F-stat; P-value	Mean ± SD	F-stat; P-value
Age group		0.8; 0.562		1.0; 0.434
18–24	34.6 ± 16.4		71.5 ± 30.0	
25–34	31.7 ± 11.5		56.4 ± 33.0	
35–44	30.1 ± 12.0		61.9 ± 29.7	
45–54	32.0 ± 12.2		61.0 ± 30.2	
>54	30.8 ± 14.0		64.2 ± 34.3	
Gender		−1.1^T^; 0.254		−6.9^T^; <0.001***
Male	30.5 ± 11.9		51.3 ± 28.7	
Female	31.9 ± 12.4		71.7 ± 29.4	
Marital status		0.4; 0.772		3.8; 0.010*
Never Married	31.7 ± 12.5		56.8 ± 41.1	
Married	31.1 ± 12.1		60.7 ± 29.3	
Widowed	31.2 ± 12.0		59.7 ± 0.0	
Divorced	27.7 ± 13.2		88.9 ± 0.0	
Religion		2.2^T^; 0.030*		−8.4^T^; <0.001***
Christian	31.8 ± 12.4		56.0 ± 31.8	
Non-Christian	28.7 ± 10.8		78.5 ± 17.8	
Highest education		9.0; <0.001***		37.5; <0.001***
Primary	29.4 ± 10.7		70.8 ± 11.0	
Junior high	29.8 ± 12.5		73.5 ± 28.8	
Senior high	31.0 ± 11.2		50.4 ± 27.6	
Tertiary	41.1 ± 13.1		27.0 ± 38.8	
Ethnicity		2.4; 0.091		7.9; <0.001***
Akan	33.7 ± 13.1		56.2 ± 35.4	
Bono	30.2 ± 11.9		58.4 ± 29.8	
Dagaaba/ Frafra/ Mo	31.2 ± 11.7		73.1 ± 25.4	
Household size		10.2; <0.001***		40.0; <0.001***
Alone	41.3 ± 12.0		0.0 ± 0.0	
<4	29.2 ± 10.5		65.1 ± 26.9	
4–5	30.2 ± 11.5		64.8 ± 27.8	
6–7	36.4 ± 16.0		61.2 ± 30.1	
Employment sector		−0.4^T^; 0.687		−33.2^T^; <0.001***
Formal	30.7 ± 10.9		18.5 ± 7.9	
Informal	31.2 ± 12.5		72.3 ± 23.8	
Monthly income		5.7; <0.001***		53.4; <0.001***
No income	41.3 ± 12.0		0.0 ± 0.0	
<500	32.2 ± 12.5		71.3 ± 26.5	
500–750	28.9 ± 11.9		65.1 ± 26.9	
750–999	30.1 ± 8.1		73.3 ± 7.6	
>999	31.1 ± 12.0		37.0 ± 25.9	
Relationship to patient		0.4; 0.745		29.1; <0.001***
Family member	31.5 ± 12.5		60.8 ± 30.7	
Friend	29.7 ± 11.7		75.0 ± 22.3	
Health worker	31.0 ± 9.8		12.0 ± 0.0	
Spouse	31.3 ± 10.0		63.2 ± 0.0	
District		6.7; <0.001***		0.1; 0.985
Berekum	30.0 ± 12.7		59.8 ± 31.1	
Dormaa	35.8 ± 13.3		63.3 ± 30.6	
Jaman North	35.5 ± 14.5		59.0 ± 32.0	
Jaman South	25.5 ± 6.2		59.8 ± 29.2	
Sunyani	28.2 ± 8.6		61.0 ± 30.9	
Tain	33.1 ± 13.5		61.5 ± 31.2	

*Note: SD: standard deviation; *: p < 0.05; **: p < 0.01; ***: p < 0.001; T: Welch's t-test value.

## 4. Discussion

This study was a cross-sectional design using cost-of-illness approach to determine the direct and indirect costs associated with treatment support in the Bono Region of Ghana. Three hundred eighty-five treatment supporters were interviewed.

The study findings revealed that 45% of the respondents were within the 35–44-year age bracket, and most (72%) of them were family members. This suggests that people who are economically active have to take some time off their daily work to do the unpaid TB treatment support work. The loss of income associated with the treatment support work could have some financial consequences not only on the treatment supporter but on the entire family as well. Oftentimes, these individuals are the breadwinners of their family.

The findings of the study highlight the importance of family solidarity in times of need for family members. It confirms what was reported in Pakistan, where most TB treatment supporters were family members and community volunteers [5]. The findings of this current study also support a review of caregivers of patients with mental illness, where it was found that most caregivers were family members [15].

### 4.1. Determinants of direct cost of treatment support to the treatment supporter

Consistent with other tuberculosis costs studies, the major components of treatment supporters' direct costs were travels and feeding [16],[17]. These and other direct costs, such as miscellaneous costs, contributed to the overall direct cost estimated. Over half (50.2%) of the direct costs were attributed to travel to and from the hospital for drug rations. Other studies also found that transportation costs constituted the single largest component of direct costs associated with TB treatment [18],[19]. The travel costs associated with TB treatment support may be attributed to the frequency, mode of transportation and distance of travel to the hospital.

In addition, tuberculosis treatment takes a long period of 6 months to be completed and requires frequent traveling from home to the hospital for anti-tuberculosis drug rations. Travel costs were also incurred to ensure that the treatment supporters move from their homes to the TB patients' residences to observe the TB patients take their daily medications. Travel costs are of particular concern to treatment supporters. These costs accumulate over time without being reimbursed.

In relation to the literature, the current findings are in line with earlier findings in Zambia [16] which indicated that, among the direct costs incurred, transportation cost was greater for caregivers than the other cost components and that these costs were incurred to enable caregivers' travel to and from the health facility. The findings of the present study also agree with the findings of a study in Ethiopia where most of the direct costs incurred from TB treatment were associated with traveling [17].

Comparing the sources of evidence of direct and indirect costs of tuberculosis from different settings, time periods, patient groups and methodological approaches could be challenging. However, several studies from various settings such as Africa and Asia using different costing approaches and target groups consistently found that the cost of tuberculosis was substantial, similar to the findings our study [20]–[23].

The findings revealed that religion, highest level of education, household size, monthly income and district of residence (p < 0.001) were independent predictors of the direct cost of providing TB treatment support. Previous studies done elsewhere, though not analyzed from the treatment supporters' perspective, revealed similar findings [10],[17],[24]. These findings align with what was found in Ethiopia, where education, income and place of residence directly influenced the direct cost of tuberculosis treatment [17].

The findings of the present study are also similar to the findings of a study where educational level, income status and place influenced the direct cost of TB treatment in Benin [10]. A study in China found that gender, education and living in a rural area directly influenced the cost of seeking TB treatment in China [24]. In addition, Asres and colleagues found that gender, formal education attainment and having rural residency were significant independent factors associated with direct costs among patients attending DOTS Centre in Ethiopia. Although the analysis was done from the TB patients' perspective, factors identified were similar to those factors revealed in the present study [25].

### 4.2. Determinants of indirect cost of treatment support to the treatment supporter

The findings of the study revealed that treatment supporters lost productive time supporting TB patients. Those in the informal sector incurred a larger component of the indirect costs associated with TB treatment support than those in the formal sector.

The indirect costs (productivity loss) or forgone work hours of all treatment supporters were valued using the Human Capital Approach as proposed by earlier analysts [13],[26]. Productivity loss was classified by sectors (formal and informal sector) of employment. Productivity loss for the informal sector was valued at the average agricultural daily wage of the Bono Region collected during the field survey. The national daily minimum wage rate was used to value the productivity hours lost by treatment supporters who were employed in the formal sector [14].

Studies related to productivity losses due to tuberculosis in other countries were compared with this current study. For instance, a study found that the indirect cost of tuberculosis to patients was US$ 528 in Nigeria [27], higher than the indirect cost estimated in this current study. The findings from Nigeria included time lost due to hospital stay, while the current study did not. In India, the estimated mean indirect cost for TB treatment was US$ 526.87 per patient [28]. Again, the indirect cost estimated in the India study was higher than what was found in the current study.

These differences could be related to the contextual factors in healthcare delivery. The cost of living and differences in costs of services in these contexts could be explained by the higher indirect cost estimates. For example, in Ghana, tuberculosis treatment is outpatient service, and tuberculosis patients and their treatment supporters do not need to stay overnight. Also, tuberculosis patients and treatment supporters have specific dates to replenish their drug, so they do not all crowd at the health care unit, which may require them to pass the night at the hospital or guest house.

The findings revealed that household size could influence the total indirect cost of assisting tuberculosis patients with treatment. This implied that treatment supporters from households with large sizes were more likely to incur indirect cost than treatment supporters with smaller household sizes. This aligns with the findings of a study which also identified households with a larger number of people as an independent predictor of the total indirect cost associated with illness care [29].

Higher level of education attainment was identified as one the determinants of indirect cost associated with tuberculosis treatment support. Treatment supporters with higher level of education have a higher likelihood of incurring less cost than those with low level of education. Relating our findings to previous studies, for instance, it was reported in Nigeria that tuberculosis patients and supporters who have higher levels of education are less likely to incur higher indirect costs compared to those who have lower levels of educational attainment [30]. Also, the level of educational attainment was identified in Benin as an independent predictor of the indirect costs of seeking tuberculosis treatment [10]. Level of educational attainment is widely recognized as a determinant of the social and economic status of individuals, as affirmed by our current study.

Furthermore, we found ethnicity of the treatment supporters to be associated with indirect costs. Treatment supporters who were Akan were highly likely to incur less indirect cost than those who were non-Akan. In Ghana, the Akan are widely recognized to be wealthier and have better social support networks as compared to other ethnic groupings.

### 4.3. Strength and limitations of the study

This study was novel as it provided estimates of costs associated with TB treatment to the treatment supporters. Most of the previous studies estimated only the costs associated with TB to the patients, household and the health system. There are a few limitations. In this study we did not use salaries in estimating the indirect costs but rather the daily minimum wage / average daily agricultural wage rate, which could have resulted in over- or under-estimation of the indirect costs. We did not account for distance to the health facility, which affects both the direct and indirect costs estimated. Furthermore, we included in our study only treatment supporters who were supporting patients registered with DOTS centers for treatment, which limits the representativeness of the costs estimates. Challenges with precise recall of treatment support related expenditures by treatment supporters could not be dealt with completely.

## 5. Conclusions

We concluded that tuberculosis treatment supporters incurred an average total direct cost of GHS 46.7 (US$ 8.7) and lost 16.3 hours amounting to GHS 65.7 (US$ 12.33) per month. These costs are significant to the treatment supporters and were incurred to ensure access to treatment, prevent treatment defaulting and enhance treatment completion.

We therefore recommend that policy should be geared toward mitigating these costs to alleviate the adverse effects on the socio-economic status and future welfare of treatment supporters. This would be progress in right direction toward achieving the End TB goal of making the world free of tuberculosis by 2035.
